# The association between previous and future severe exacerbations of chronic obstructive pulmonary disease: Updating the literature using robust statistical methodology

**DOI:** 10.1371/journal.pone.0191243

**Published:** 2018-01-19

**Authors:** Mohsen Sadatsafavi, Hui Xie, Mahyar Etminan, Kate Johnson, J. Mark FitzGerald, for the Canadian Respiratory Research Network

**Affiliations:** 1 Respiratory Evaluation Sciences Program, Faculty of Pharmaceutical Sciences, the University of British Columbia, Vancouver, Canada; 2 Institute for Heart and Lung Health, Faculty of Medicine, the University of British Columbia, Vancouver, Canada; 3 Centre for Clinical Epidemiology and Evaluation, the University of British Columbia, Vancouver, Canada; 4 Biostatistics Division, Faculty of Health Sciences, Simon Fraser University, Burnaby, Canada; National and Kapodistrian University of Athens, SWITZERLAND

## Abstract

**Background:**

There is minimal evidence on the extent to which the occurrence of a severe acute exacerbation of COPD that results in hospitalization affects the subsequent disease course. Previous studies on this topic did not generate causally-interpretable estimates. Our aim was to use corrected methodology to update previously reported estimates of the associations between previous and future exacerbations in these patients.

**Methods:**

Using administrative health data in British Columbia, Canada (1997–2012), we constructed a cohort of patients with at least one severe exacerbation, defined as an episode of inpatient care with the main diagnosis of COPD based on international classification of diseases (ICD) codes. We applied a random-effects 'joint frailty' survival model that is particularly developed for the analysis of recurrent events in the presence of competing risk of death and heterogeneity among individuals in their rate of events. Previous severe exacerbations entered the model as dummy-coded time-dependent covariates, and the model was adjusted for several observable patient and disease characteristics.

**Results:**

35,994 individuals (mean age at baseline 73.7, 49.8% female, average follow-up 3.21 years) contributed 34,271 severe exacerbations during follow-up. The first event was associated with a hazard ratio (HR) of 1.75 (95%CI 1.69–1.82) for the risk of future severe exacerbations. This risk decreased to HR = 1.36 (95%CI 1.30–1.42) for the second event and to 1.18 (95%CI 1.12–1.25) for the third event. The first two severe exacerbations that occurred during follow-up were also significantly associated with increased risk of all-cause mortality. There was substantial heterogeneity in the individual-specific rate of severe exacerbations. Even after adjusting for observable characteristics, individuals in the 97.5th percentile of exacerbation rate had 5.6 times higher rate of severe exacerbations than those in the 2.5th percentile.

**Conclusions:**

Using robust statistical methodology that controlled for heterogeneity in exacerbation rates among individuals, we demonstrated potential causal associations among past and future severe exacerbations, albeit the magnitude of association was noticeably lower than previously reported. The prevention of severe exacerbations has the potential to modify the disease trajectory.

## Introduction

Periods of sustained worsening of respiratory symptoms, referred to as acute exacerbations, are a major source of morbidity and mortality in chronic obstructive pulmonary disease (COPD)[1]. COPD is a leading cause of hospitalization in Canada due to the high prevalence of the disease and the high incidence of severe exacerbations requiring inpatient care[2]. Understanding the patterns of occurrence of exacerbations at the individual level will enable objective quantification of the long-term benefits associated with the prevention of exacerbations, resulting in more effective management strategies.

In this context, a critical question is whether the occurrence of an acute exacerbation causally affects the risk of subsequent exacerbations. Previous studies have addressed this topic, but there are important methodological issues that jeopardize the interpretability of their results. A Canadian study of more than 70,000 COPD patients in administrative health databases used a non-parametric survival model to estimate a 190% increase in the risk of future exacerbations following the first severe exacerbation during follow-up[3]. Subsequent severe exacerbations also increased the risk but there was a diminishing effect as the number of exacerbations increased. However, as explained in detail elsewhere, the hazard ratios from this study cannot be interpreted causally because the authors did not consider between-individual variability (heterogeneity) in exacerbation rates[4]. In a previous work, we have shown that in the presence of heterogeneity in the rate of exacerbations, positive associations (hazard ratios of more than one) would be observed even under the null hypothesis that current exacerbations do not affect the risk of subsequent ones[4]. As such, the literature needs to be updated with a proper evaluation of the extent to which the occurrence of exacerbations affects the risk of subsequent ones.

Fortunately, a class of survival analysis techniques can account for this heterogeneity statistically, which enables inference on the associations between recurrent exacerbations within individuals[5]. The aim of this study was to use these methods to provide a more accurate characterization of the natural history of COPD exacerbations. Our primary objective was to update the previously reported estimates of associations among severe COPD exacerbations using more robust methodology. In particular, we tested the hypothesis that the occurrence of a severe exacerbation increases the risk of subsequent severe exacerbations, and compared the magnitude of this risk as the total number of exacerbations increased. Our secondary objective was to update the previously reported estimates of associations between severe COPD exacerbations and long-term COPD mortality also using more robust methodology. Our tertiary objective was to quantify heterogeneity in exacerbation rates over and above the variability that can be attributed to observed patient and disease characteristics.

## Methods

This was a retrospective cohort study of a population-based sample of COPD patients with at least one instance of severe COPD exacerbation requiring hospital admission.

### Study subjects

British Columbia, a Canadian province with a population of 4.6 million (as of 2011[6]), hosts centralized databases that capture the health-care encounters of all legal residents regardless of their demographic characteristics or their enrolment in third party insurance programs. The quality of the data is considered very high, with low levels of missing or incorrect information[7,8]. We had access to anonymized data from the following databases for the period of 1997 to 2012: demographic and consolidation (containing demographic characteristics and longitudinal registration status with the health care system)[9], census (containing socioeconomic status [SES] quintiles for each patient-year according to neighborhood income), vital statistics-deaths[10], and inpatient[11] and outpatient[12] encounters. Ethics approval was obtained from the Human Ethics Board of the University of British Columbia (H13-00684). All inferences, opinions, and conclusions drawn in this research are those of the authors, and do not reflect the opinions or policies of the Data Steward(s).

### Study design

From these data, we created a cohort of COPD patients 35 years or older with an incident severe exacerbation (referred to as the *index* exacerbation). Severe exacerbation was defined as an episode of hospitalization with COPD as the main (most responsible) diagnostic code using the international classification for disease codes (ICD, 9th version codes 491, 492, 493.2x, 496, or ICD, 10th version codes J41, J42, J43, and J44). An incident exacerbation occurred following at least 5 years of presence in the data without the occurrence of a severe exacerbation. This period was extended to 8 years in a sensitivity analysis. We did not use additional criteria for inclusion because hospitalizations with COPD as the main diagnosis are highly predictive of having COPD[13], and because in a large proportion of COPD patients the primary diagnosis of COPD is made when a patient presents with their first severe exacerbation (with no prior COPD-related resource use)[14]. Individuals were followed until death, de-registration from the health-care system, the date of the last resource use of any type, or the end of study period (2012/12/31), whichever occurred first. Exacerbations that occurred consecutively without an intervening time interval (for example, readmission on the date of discharge) were considered the same event. Exact date of admissions and discharges were available. Due to privacy issues, death date was available by the year and month but not by the day. In the main analysis, we considered the day of last resource use of any type within the death month as the exact day of death; alternative scenarios that considered first and last day of the month as the date of death were explored in sensitivity analyses.

### Statistical analysis

We used a parametric joint (shared) frailty model adapted from the model proposed by Liu et. al. [15], and Huang et. al.[16], to model the periods between consecutive severe exacerbations with the competing risk of death. We modeled heterogeneity in the intrinsic rate of severe exacerbations between patients using random-effects terms, which represents the propensity of each individual to exacerbate or die over and above the variability due to their observed characteristics[17]. These random-effects remove the potential for confounding by any unmeasured, time-fixed covariate, and enable within-individual inference on the association between exacerbations (or between exacerbations and subsequent mortality), which is the main advantage of joint-frailty models over conventional survival models. For example, in conventional survival analysis the association between the fourth exacerbation and subsequent exacerbations is determined by comparing the time to next exacerbation in patients with at least four exacerbations versus the average between-exacerbation time in the whole sample. Even in the absence of causal associations between exacerbations, between-exacerbation time is expected to be shorter in the subgroup of patients with many exacerbations than in those with few exacerbations due to intrinsic differences between these patients. This will cause spurious positive associations between exacerbations and risk of subsequent ones in conventional survival analysis. The joint frailty model used here uses random-effects terms to capture the heterogeneity in exacerbations rates that is due to intrinsic difference between patients over and above their observed characteristics, thus removing the afore-mentioned bias. A by-product of using such random-effect terms is the quantification of between-individual variability (heterogeneity) down to the individual level.

As described in detail in the Supporting Information S1 Text, our model incorporated two proportional-hazards recurrent-event survival models, one for the hazard of severe exacerbations and one for death. The potential correlation between the exacerbation and death processes (causing death to be a competing risk for exacerbation) is captured through a multiplier for the random-effect term in the regression equation for the hazard of death.

Parametric survival models require the specification of a baseline survival function[5]. We evaluated the fit of the Weibull, log-normal, and log-logistic functions using Akaike’s Information Criterion (AIC, lower scores are better), and through a visual comparison of the predicted between-event times and the empirical Kaplan-Meier estimates. Results of model selection are provided in S1 Fig.

The follow-up time for each individual was divided into intervals comprised of the time from the end of a severe exacerbation to the beginning of the next severe exacerbation, end of follow-up, or death. The discharge date was used to define the end of a severe exacerbation (i.e., start of the time interval) with the exception of intervals that ended in death. The start of these intervals was the admission date for the previous exacerbation, as the patient was at risk for death during the inpatient period.

We included age at the beginning of each interval between severe exacerbations, calendar year at the beginning of each interval, sex, SES (defined as neighborhood income quintile) at baseline, and comorbidity (defined as the Charlson comorbidity index[18] and estimated in the 12-month period immediately before the index date) as the independent variables in the regression equations for severe exacerbation and death. In the regression equation for severe exacerbations, we also included a variable indicating whether the length of hospital stay for the index exacerbation was seven days or longer, and whether the index exacerbation required intensive care unit (ICU) care. These variables were not included in the equation for death as they were ascertained during the at-risk period and their inclusion would have resulted in immortal time bias. We defined the number of severe exacerbations during the follow-up period as a dummy-coded time-dependent covariate in both regression equations in order to evaluate the impact of the number of previous severe exacerbations on the risk of future severe exacerbations and on death. Dummy-coding of this variable was performed for the first five severe exacerbations during follow-up, with a sixth category representing all subsequent severe exacerbations combined.

Our results are presented as hazard ratios (HR) for the associations between the covariates and the risk of a future severe exacerbation or death. We quantified heterogeneity in exacerbation rates by calculating an individual-specific HR, which was derived from each individual's estimated random-effect term, and captures the individual’s propensity to exacerbate over and above the variability captured by the observed covariates. All analyses were performed in SAS version 9.4 (SAS Institute, Cary, NC). The regression was fitted using the non-linear mixed model routine (PROC NLMIXED) as described in Lu et. al.[19] The effect of modifications of the study design on the results was evaluated through sensitivity analyses (S1 Table). The SAS code is provided (S1 File).

## Results

Among all individuals 35 years or older with at least one severe exacerbation after 5 or more years of presence in the data, 169 had invalid death dates, and 13 had missing SES variables. After removing these, 35,994 individuals contributing 115,714 patient-years (average follow-up: 3.21 years) remained. These individuals contributed a total of 34,271 severe exacerbations during follow-up, corresponding to a rate of 0.30/patient-year. 18,699 patients died during follow-up, at rate of 0.16/patient-year. Table 1 provides the baseline characteristics of the final sample.

**Table 1:** Baseline characteristics of the sample, which is composed of COPD patients 35 years or older who were admitted to hospital with an incident severe exacerbation. Data are shown as mean (standard error) unless otherwise stated.

Parameter	Value
**Number of patients**	35,994
**Follow-up time (in years)**	3.21
**% female**	49.8%
**Age at baseline (in years)**	73.75 (11.83)
**Charlson comorbidity index**	2.50 (1.82)
**Cohort year (in years since 1/1/2000)**	6.90 (3.34)
**Number in socio-economic status**	
Low	19,154 (53.2%)
High	16,100 (44.7%)
Missing	740 (2.1%)
**Severe exacerbations**	
Total number	34,271
Rate	0.30
**Frequency of follow-up severe exacerbations**	
0	22,146 (61.5%)
1	6,912 (19.2%)
2	2,872 (8.0%)
3	1,472 (4.1%)
4	892 (2.5%)
5	526 (1.5%)
6+	1,174 (3.3%)
**Death**	
Total number	18,699
Annual rate	0.16

Among the three models examined, the Weibull model was the best fit to the shape of the survival curve for the period between a previous severe exacerbation and the next severe exacerbation or death (S1 Fig). It was also the most supported model using AIC (S1 Table).

### Factors associated with risk of severe exacerbations

**Fig 1:**
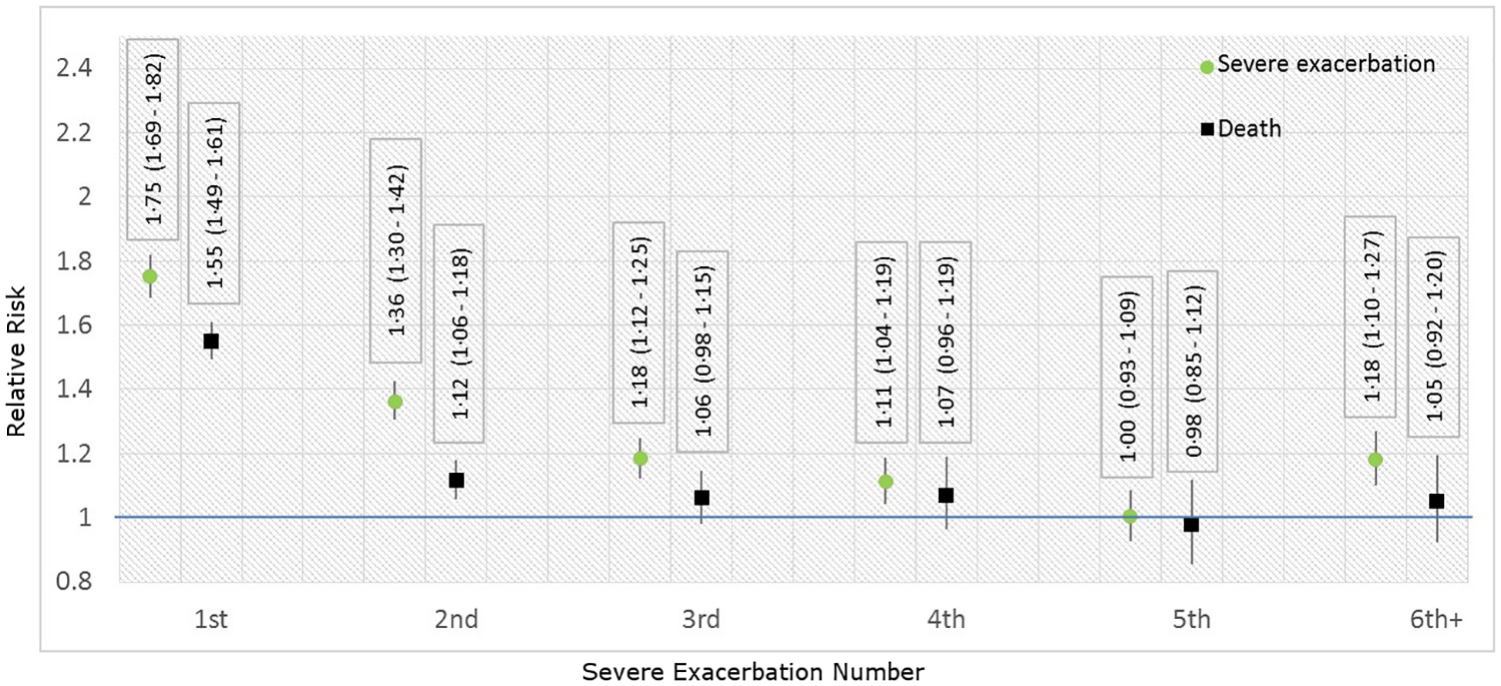
Regression coefficients relating the occurrence of each follow-up severe exacerbations to subsequent severe exacerbations (green circles) or death (black squares). **Footnote:** Regression coefficients for all variables are provided in S2 Table. For each exacerbation, the reference (baseline) hazard for the reported HR is the period between the immediately previous and the current exacerbation.

Fig 1 provides the results from the joint frailty model. The hazard ratios (HRs) represent the effect of each severe exacerbation during follow-up on the risk of subsequent severe exacerbations. For each exacerbation, the reference (baseline) hazard for the reported HR was the preceding time interval, consisting of the period between the previous exacerbation and the current exacerbation.

There was a positive but diminishing association between each severe exacerbation and the risk of future exacerbations. The first exacerbation during follow-up was associated with an increased risk of 75% (HR = 1.75, 95%CI 1.69–1.72, P<0.001). The second severe exacerbation during follow-up was associated with a further increased risk of 36% (HR = 1.36, 95%CI 1.30–1.42, P<0.001); the third and fourth exacerbations were associated with further increased risks of 18% (HR = 1.18 95%CI 1.12–1.25, P = 0.017) and 11% (HR = 1.11, 95%CI 1.04–1.19, P<0.001), respectively. These HRs are multiplicative, therefore the risk of severe exacerbation was 3.14 times higher after the fourth severe exacerbation during follow-up than after the index severe exacerbation. The coefficient for the fifth severe exacerbation was not statistically significant, but the combined effect of the sixth and all subsequent severe exacerbations was statistically significant (HR = 1.18, 95%CI 1.10–1.27, P<0.001).

The first two severe exacerbations during follow-up were significantly associated with an increased risk of all-cause mortality. The first follow-up exacerbation was associated with an increased risk of 55% (HR = 1.55, 95%CI 1.49–1.61); this was reduced to 12% (HR = 1.12, 95%CI 1.06–1.18) for the second event. Subsequent severe exacerbations were not associated with a change in the risk of mortality. Regression coefficients for all included variables are provided in S2 Table.

### Heterogeneity in the rate of severe exacerbations

**Fig 2:**
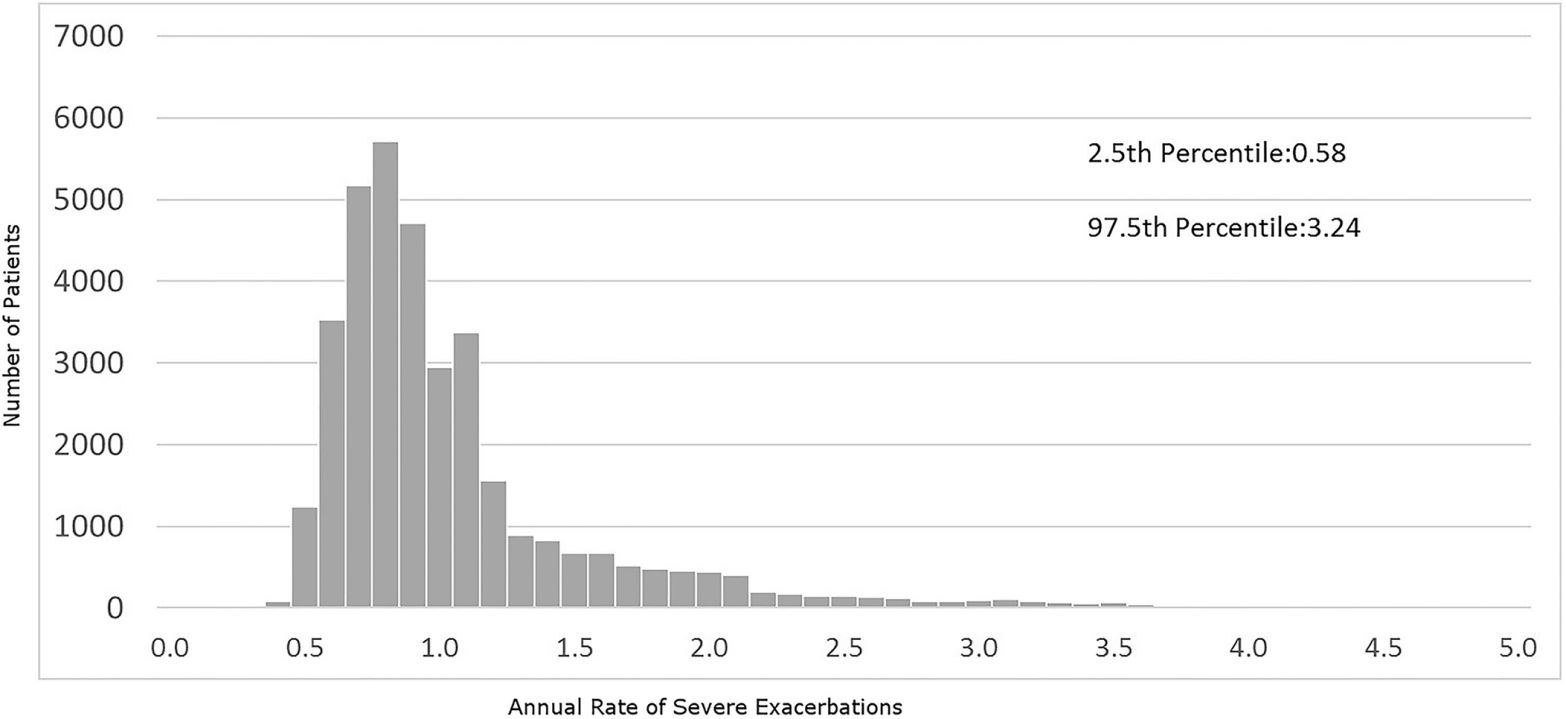
Individualized hazard ratios* of severe exacerbations after removing the effects of observed patient characteristics†. **Footnote:** * Individual-specific HRs represent the tendency of individuals to exacerbate or die that exceeds the effects of the independent variables included in the model. † 0.8% of individuals had HRs of greater than 5 and were not shown in this graph.

Fig 2 presents the distribution of individualized HRs for severe exacerbations. 95% of individuals had a HR between 0.58 and 3.24, meaning that individuals in the 97.5^th^ percentile had 5.6 times higher rate of severe exacerbations than those in the 2.5^th^ percentile after adjusting for their observed characteristics.

### Sensitivity analysis

The results of the sensitivity analyses are provided in S3, S4 and S5 Tables. In general, there were no major changes to our findings on the associations between past and future severe exacerbations and their relation to death, and the magnitude of the HRs remained largely the same.

## Discussion

The main purpose of this study was to update the previously-reported associations[3] between recurrent severe COPD exacerbations using more robust statistical methodology. We found a positive but diminishing effect of severe exacerbations on the risk of future exacerbations in a large population-based cohort. The first severe exacerbation during follow-up was associated with a 75% increase in the risk of subsequent severe exacerbations, which was reduced to 11% for the fourth severe exacerbation. Although other investigators have examined the associations between exacerbations within the same individual[3], they used conventional survival analysis techniques that did not account for the severe confounding effect of heterogeneity in exacerbation rates between individuals[4]. While we have reached similar overall conclusions, the magnitude of our estimates are very different from the previously reported estimates[20]. The HR for the first severe exacerbation during follow-up was 1.75 in this analysis, compared with 2.90 in the analysis by Suissa et. al.[3], and the combined effect of the first four severe exacerbations during follow-up was 3.14 in our study, versus 6.90 in Suissa et. al.

The difference in the previously-reported associations between exacerbations and those reported here can have substantial implications for estimating the long-term benefits of strategies aimed at preventing or delaying exacerbations in COPD patients. For example, preventive therapy with azithromycin (a broad-spectrum antibiotic) is associated with a 27% reduction in exacerbation rates[21]. The positive association between severe exacerbations means that even after discontinuation of azithromycin, patients might experience a lower rate of exacerbations than their counterparts who did not receive preventive therapy (due to fewer exacerbations experienced while on preventive therapy). A cost-effectiveness analysis that ignores the positive association between exacerbations will underestimate the value of preventive therapy, whereas a cost-effectiveness analysis that uses the previously-reported estimates of these associations is likely to over-estimate the benefit of therapy. We also demonstrated substantial between-individual variability (heterogeneity) in severe exacerbation rates, which suggests that the outcomes of treatment and prevention strategies might also vastly vary among patients. Predictors (e.g., biomarkers) that identify where a patient belongs on the spectrum of exacerbation risk will be instrumental in designing patient-centered preventive approaches for COPD exacerbations.

We observed a positive association between exacerbations despite controlling for individual heterogeneity in exacerbation rates. This suggests that there are potentially causal associations between severe exacerbations. Causal associations may be due exacerbation-induced structural alterations in the lung, such as a loss of tissue elasticity due to an increased level of inflammatory mediators (tumor necrosis factor alpha, interleukin 6)[1]. Acute inflammation triggers the initiation of repair mechanisms and might result in permanent tissue remodeling. The effects of this remodeling might present as airway obstruction or a reduction in health status (manifested as breathlessness or other respiratory or general symptoms)[22]. Indeed, the duration and extent of health status decline after an exacerbation seem to be disproportionate compared with the observed decline in airway obstruction, suggesting that there is damage beyond what is indicated by the decline in lung function[22]. However, this study was not designed to examine the mechanism of the observed associations. A natural extension of this research is to evaluate multiple markers representing inflammation, lung function, and health status to better understand the degree to which different pathways are responsible for the long-term effects of exacerbations.

To the best of our knowledge, this study is the first to use frailty models to determine the association between severe COPD exacerbations. These models allow heterogeneity in exacerbation rates to be quantified at the individual level, and provide more credible evidence for the potential causal associations between exacerbations. Previous studies have reported prediction equations for the expected (average) rate of exacerbations for patients according to their characteristics[23]. Frailty models can be used in conjunction with such prediction tools to make probabilistic predictions of the individualized rate and timing of future exacerbations (along with measures of uncertainty such as 95% prediction intervals)[24]. These predictions are highly clinically relevant and can be used to communicate not only the expected outcomes but the uncertainty and risk and can be used as shared decision making tools between patients and care providers for more informed decisions.

This study also has important limitations. Reliance on the administrative databases means our estimates are only accurate to the level of precision in the diagnostic coding of severe exacerbations. However, previous chart review studies have shown the coding to be relatively accurate[13]. We focused on severe exacerbations requiring inpatient care. While there are algorithms for identifying mild or moderate exacerbations in the administrative data, we believe exacerbations requiring hospitalisation are most reliably captured in the data, and have the highest potential to affect the course of COPD. We did not have access to other important covariates such as lung function measures, smoking status, or functional impartment that could potentially be associated with exacerbation risk. Incorporating these variables could provide information on mediating pathways, such as the degree to which the increased risk of future exacerbations is channeled through a reduction in lung function. Further, exacerbations are a heterogeneous group of events (a recent cluster analysis identified several distinct subgroups[25]), and a more nuanced analysis that incorporates phenotypic information could further elucidate the mechanistic pathways of the impact of exacerbations on the lungs.

## Conclusion

Our study suggests that there is substantial heterogeneity in the rate of severe COPD exacerbations among individuals, and that there is a positive association between the occurrence of severe exacerbations and the risk of subsequent severe exacerbations. From a methodological perspective, our study demonstrates the utility of frailty models, which are a robust and well-established statistical framework for modeling recurrent events and are under-utilized in respiratory research[24]. From a clinical and policy perspective, the degree to which exacerbations affect the natural history of COPD is key to evaluating the merits of preventive exacerbation therapies. Further research using more enriched datasets that include markers of disease activity can help us better understand the disease mechanisms and formulate more efficient disease management strategies to combat the high and escalating burden of COPD.

## Supporting information

S1 Text Details of the joint frailty model. (DOCX)

S1 Fig Comparing model fits. (DOCX)

S1 Table Comparing model fits. (DOCX)

S2 Table Regression coefficients for the joint frailty model. (DOCX)

S3 Table Results of the sensitivity analysis: Eight-year wash-in period. (DOCX)

S4 Table Results of the sensitivity analyses: Death defined as the first day of the month that death occurred. (DOCX)

S5 Table Results of the sensitivity analyses: Death defined as the last day of the month that death occurred. (DOCX)

S1 File SAS code for the analyses. (TXT)

## Abbreviations

AIC: Akaike Information Criterion
CI: Confidence Interval
COPD: Chronic obstructive pulmonary disease
ICD: International Classification of Disease
HR: Hazard ratio
