# Supplementary material for: The association between previous and future severe exacerbations of chronic obstructive pulmonary disease: Updating the literature using robust statistical methodology
Source: PLoS One. 2018 Jan 19;13(1):e0191243. doi: 10.1371/journal.pone.0191243 (PMC5774719; doi:10.1371/journal.pone.0191243)
Supplement: S1 Text — (DOCX) [file pone.0191243.s001.docx]

Supplementary material for the manuscript

Between-individual variability and within-individual associations in severe exacerbations of COPD

**Authors:** Mohsen Sadatsafavi; Hui Xie; Mahyar Etminan; J Mark FitzGerald; *for the Canadian Respiratory Research Network*

# S1: Details of the shared frailty model

The framework proposed by Lui et. al.^1^ is for the analysis of correlated recurrent events and a terminal event. Here, the recurrent events are severe COPD exacerbations, and the terminal event is death. The setup is as follows: the index date (t=0) is the date of discharge from hospital for the first severe COPD exacerbation. Each subsequent severe exacerbation (indexed 1,2,3, …) is identified by its first day of hospital admission since the index date. Follow-up ends at date of death, or else at the last dat of follow-up.

Using the notation by Liu et. al., we have

Rate of exacerbation $r_{i}\left( t \right)=\exp\left( \beta^{T}.X_{i}+v_{i} \right).r_{0}(t)$

Rate of death $\lambda_{i}\left( t \right)=\exp\left( {\beta'}^{T}.X_{i}+{\gamma.v}_{i} \right).\lambda_{0}(t)$

for the *i^th^* patient, where β are coefficients of observed covariates on the severe exacerbation rate, β’ are coefficients of observed covariates on mortality, and $r_{0}(t)$and $\lambda_{0}(t)$ are baseline hazards for recurrent (severe COPD exacerbations) and terminal (death) event processes, respectively. $X_{i}$ is the set of covariates for the *i^th^* patient. $v_{i}$ is the individual-specific random-effects term that captures heterogeneity in the exacerbation rate that is left unexplained by observed covariates. $\gamma$ is the parameter that results in 'sharing' the random-effect term between the exacerbation and death hazards. A $\gamma=0$ makes the death process to be completely independent of the severe exacerbation process, conditional on covariates. The correlation between these two processes is therefore introduced by ${\gamma.v}_{i}$. In the implementation of this model in SAS^2^, Lui et. al. used piecewise constant functions for the hazard. Here, we examined log-normal, log-logistic, and Weibull survival functions (each giving rise to a unique hazard function). The best fit was from the Weibull model, with the hazard functions being of the form^3^

$$r_{0}(t)=\delta.\alpha.{(\alpha.t)}^{\delta-1}$$

for exacerbation, and a similar hazard function

$$\lambda_{0}(t)=\delta'.\alpha'.{(\alpha'.t)}^{\delta'-1}$$

for mortality.

One of the covariates ($X_{i}$) included in both the rate and mortality regression models was the dummy-coded number of previous exacerbations. This enables capturing the adjusted effect of the occurrence of each severe exacerbation on the rate of subsequent exacerbations or the risk of death.

The SAS non-linear mixed model procedure (PROC NLMIXED) was used to find out the maximum likelihood estimates of the parameters. The data are set up as ‘gap time’: each row belongs to gap times between events for each patients, with the first row representing the time interval between the index date to the admission date of the first follow-up severe exacerbation, the second row from the discharge date of the first follow-up COPD exacerbation to the admission date of the third follow-up severe exacerbations, and so on. The last interval ends at date of death or censoring. Note that for the interval that ended up in death, the start of the interval was moved back to the admission date of the last severe exacerbation (rather than its discharge date). This is because while patients during an episode of admission for severe exacerbation are not at risk of another severe exacerbation, they remain at risk of death.

As described in Lui et. al., the model requires that recurrent, terminating, and censoring processes all have continuous distribution and censoring to be non-informative.

The SAS code is available from the authors upon request.

**References**

1. Liu L, Wolfe RA, Huang X. Shared frailty models for recurrent events and a terminal event. Biometrics. 2004 Sep;60(3):747–56.

2. Lu L, Liu C. Analysis of Correlated Recurrent and Terminal Events Data in SAS [Internet]. NorthEast SAS Users Group (NESUG); 2008 [cited 2015 Oct 16]. Available from: http://www.lexjansen.com/nesug/nesug08/sa/sa16.pdf

3. Carroll KJ. On the use and utility of the Weibull model in the analysis of survival data. Control Clin Trials. 2003 Dec;24(6):682–701.
