# Supplementary material for: The association between previous and future severe exacerbations of chronic obstructive pulmonary disease: Updating the literature using robust statistical methodology
Source: PLoS One. 2018 Jan 19;13(1):e0191243. doi: 10.1371/journal.pone.0191243 (PMC5774719; doi:10.1371/journal.pone.0191243)
Supplement: S1 Fig — (DOCX) [file pone.0191243.s002.docx]

Supplementary material for the manuscript

Between-individual variability and within-individual associations in severe exacerbations of COPD

**Authors:** Mohsen Sadatsafavi, PhD^1,2,3^; Hui Xie, PhD^4^; Mahyar Etminan, PharmD^2^; J Mark FitzGerald, MD^2,3^; *for the Canadian Respiratory Research Network*

# S1 Fig: Comparing model fits

| **Figure A2.1:** The Kaplan-Meier curve and the parametric estimates for time to next severe exacerbation or death |
| --- |
| **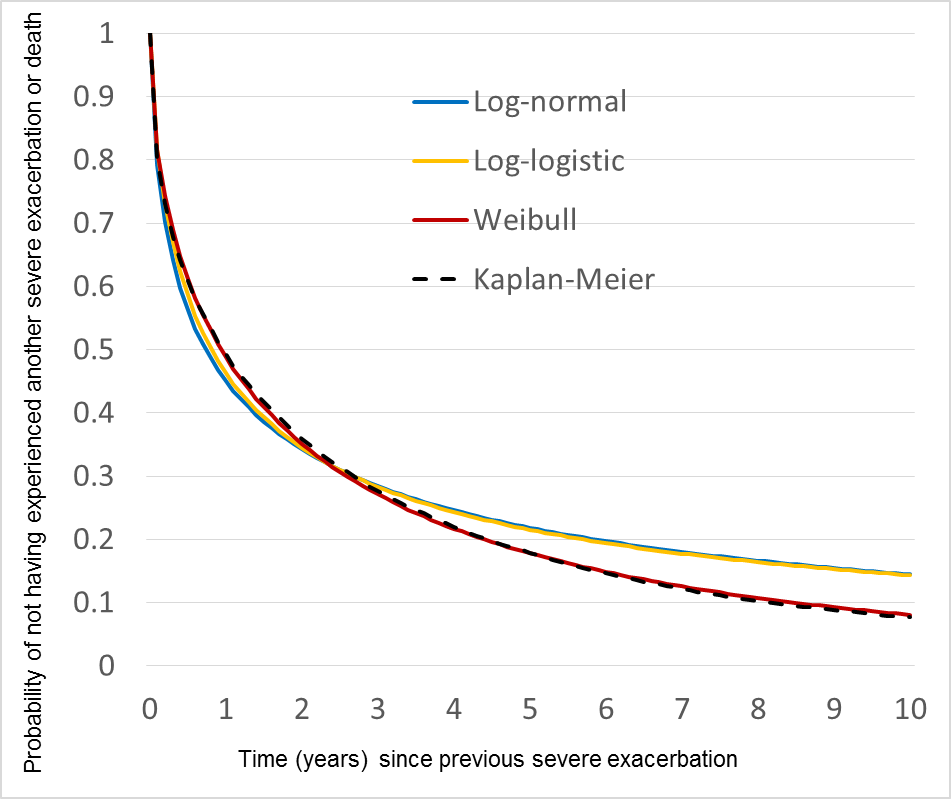** |
|  |
