# Supplementary material for: The association between previous and future severe exacerbations of chronic obstructive pulmonary disease: Updating the literature using robust statistical methodology
Source: PLoS One. 2018 Jan 19;13(1):e0191243. doi: 10.1371/journal.pone.0191243 (PMC5774719; doi:10.1371/journal.pone.0191243)
Supplement: S1 Table — (DOCX) [file pone.0191243.s003.docx]

Supplementary material for the manuscript

Between-individual variability and within-individual associations in severe exacerbations of COPD

**Authors:** Mohsen Sadatsafavi; Hui Xie; Mahyar Etminan; J Mark FitzGerald; *for the Canadian Respiratory Research Network*

# S1 Table: Regression coefficients for the joint frailty model

| **Table 2:** Hazard ratio (HR) for the final model | | | | | | | | |
| --- | --- | --- | --- | --- | --- | --- | --- | --- |
|  | **Exacerbation** | | | | **Mortality** | | | |
| **Parameter** | **HR** | **95% CI** | **P** | **HR** | | **95% CI** | **P** |  |
| **Exacerbation 1** | 1.75 | 1.69 , 1.82 | <0.001* | 1.55 | | 1.49 , 1.61 | <0.001* |  |
| **Exacerbation 2** | 1.36 | 1.30 , 1.42 | <0.001* | 1.12 | | 1.06 , 1.18 | <0.001* |  |
| **Exacerbation 3** | 1.18 | 1.12 , 1.25 | <0.001* | 1.06 | | 0.98 , 1.15 | 0.148 |  |
| **Exacerbation 4** | 1.11 | 1.04 , 1.19 | 0.001* | 1.07 | | 0.96 , 1.19 | 0.209 |  |
| **Exacerbation 5** | 1.00 | 0.93 , 1.09 | 0.942 | 0.98 | | 0.85 , 1.12 | 0.742 |  |
| **Exacerbation 6+** | 1.18 | 1.10 , 1.27 | <0.001* | 1.05 | | 0.92 , 1.20 | 0.450 |  |
| **Other covariates** | | | | | | | |  |
| Sex (female v. male) | 0.83 | 0.81 , 0.85 | <0.001* | 0.85 | | 0.83 , 0.88 | <0.001* |  |
| Age at baseline (per 10 years increase) | 1.08 | 1.07 , 1.10 | <0.001* | 1.59 | | 1.56 , 1.61 | <0.001* |  |
| Charlson comorbidity index | 1.02 | 1.01 , 1.03 | <0.001* | 1.19 | | 1.18 , 1.19 | <0.001* |  |
| Cohort year | 0.99 | 0.99 , 1.00 | 0.002* | 0.98 | | 0.97 , 0.98 | <0.001* |  |
| SES (high v. low) | 0.77 | 0.74 , 0.79 | <0.001* | 1.05 | | 1.02 , 1.08 | 0.002* |  |
| SES (missing v. low) | 0.60 | 0.53 , 0.68 | <0.001* | 1.45 | | 1.31 , 1.61 | <0.001* |  |
| Long length of stay at baseline | 1.08 | 1.04 , 1.13 | <0.001* | #N/A | | #N/A | #N/A |  |
| If the patient was admitted to ICU | 1.06 | 1.02 , 1.11 | 0.008* | #N/A | | #N/A | #N/A |  |
| **Significant at 0.05 level*  *#Not included in the regression for death because it is ascertained during the follow-up time and can be associated with immortal time bias.* | | | | | | | |  |
