# Supplementary material for: The association between previous and future severe exacerbations of chronic obstructive pulmonary disease: Updating the literature using robust statistical methodology
Source: PLoS One. 2018 Jan 19;13(1):e0191243. doi: 10.1371/journal.pone.0191243 (PMC5774719; doi:10.1371/journal.pone.0191243)
Supplement: S2 Table — (DOCX) [file pone.0191243.s004.docx]

Supplementary material for the manuscript

Between-individual variability and within-individual associations in severe exacerbations of COPD

**Authors:** Mohsen Sadatsafavi; Hui Xie; Mahyar Etminan; J Mark FitzGerald; *for the Canadian Respiratory Research Network*

# S2 Table: Comparing model fits based on Akaike Information Criterion

|  | Weibull | Log-normal | Log-logistic |
| --- | --- | --- | --- |
| Akaike Information Criterion (AIC) | 134,206 | 137,964 | 138,648 |
