# Supplementary material for: The association between previous and future severe exacerbations of chronic obstructive pulmonary disease: Updating the literature using robust statistical methodology
Source: PLoS One. 2018 Jan 19;13(1):e0191243. doi: 10.1371/journal.pone.0191243 (PMC5774719; doi:10.1371/journal.pone.0191243)
Supplement: S3 Table — (DOCX) [file pone.0191243.s005.docx]

Supplementary material for the manuscript

Between-individual variability and within-individual associations in severe exacerbations of COPD

**Authors:** Mohsen Sadatsafavi; Hui Xie; Mahyar Etminan; J Mark FitzGerald; *for the Canadian Respiratory Research Network*

# S3 Table: Results of the sensitivity analysis: Eight-year wash-in period

In this analysis, we extended the required ‘wash-in’ period, the period before the index COPD hospitalization in which the patient had to be present in the data, from 5 to 8 years. The wash-in period criterion was imposed to ensure the index COPD-related hospitalization is truly the first event for each patient. This analysis results in 30,711 final patients, average follow-up of 2.91 years, 26,615 AECOPDs during the follow-up period, and 14,834 deaths

***Table E1: regression analysis results***

| **Parameter** | **HR** | **95% CI** | **P** | **HR** | **95% CI** | **P** |
| --- | --- | --- | --- | --- | --- | --- |
| **Gamma** |  |  |  |  |  |  |
| **Sex (female . Male)** | 0.83 | 0.80 , 0.86 | <0.001* | 0.84 | 0.81 , 0.87 | <0.001* |
| **Age at baseline** | 1.07 | 1.06 , 1.09 | <0.001* | 1.58 | 1.55 , 1.61 | <0.001* |
| **Charlson comorbidity index** | 1.02 | 1.01 , 1.03 | <0.001* | 1.19 | 1.17 , 1.20 | <0.001* |
| **Cohort year** | 0.99 | 0.99 , 1.00 | 0.143 | 0.97 | 0.96 , 0.98 | <0.001* |
| **SES (high v. low)** | 0.79 | 0.76 , 0.82 | <0.001* | 1.01 | 0.98 , 1.05 | 0.507 |
| **SES (missing v. low)** | 0.69 | 0.60 , 0.81 | <0.001* | 1.26 | 1.09 , 1.46 | 0.002* |
| **Long length of stay at baseline** | 1.06 | 1.01 , 1.11 | 0.012* | #N/A | #N/A | #N/A |
| **If the patient was admitted at ICU** | 1.08 | 1.02 , 1.14 | 0.009* | #N/A | #N/A | #N/A |
| **Exacerbation 1** | 1.77 | 1.69 , 1.86 | <0.001* | 1.59 | 1.52 , 1.66 | <0.001* |
| **Exacerbation 2** | 1.33 | 1.26 , 1.41 | <0.001* | 1.04 | 0.97 , 1.11 | 0.303 |
| **Exacerbation 3** | 1.23 | 1.14 , 1.31 | <0.001* | 1.00 | 0.91 , 1.12 | 0.931 |
| **Exacerbation 4** | 1.11 | 1.03 , 1.21 | 0.011* | 1.09 | 0.95 , 1.25 | 0.239 |
| **Exacerbation 5** | 1.03 | 0.93 , 1.14 | 0.565 | 0.97 | 0.81 , 1.16 | 0.729 |
| **Exacerbation 6+** | 1.17 | 1.06 , 1.29 | 0.001* | 1.03 | 0.86 , 1.22 | 0.775 |
