# Supplementary material for: The association between previous and future severe exacerbations of chronic obstructive pulmonary disease: Updating the literature using robust statistical methodology
Source: PLoS One. 2018 Jan 19;13(1):e0191243. doi: 10.1371/journal.pone.0191243 (PMC5774719; doi:10.1371/journal.pone.0191243)
Supplement: S4 Table — (DOCX) [file pone.0191243.s006.docx]

Supplementary material for the manuscript

Between-individual variability and within-individual associations in severe exacerbations of COPD

**Authors:** Mohsen Sadatsafavi; Hui Xie; Mahyar Etminan; J Mark FitzGerald; *for the Canadian Respiratory Research Network*

# S4 Table: Results of the sensitivity analyses: Death defined as the first day of the month that death occurred

Due to privacy concerns, the date of death is available by year and month but not by date. In the main analysis, we considered the day of last resource use of any type within the death month as the exact day of death. In this sensitivity analysis, we assumed the date of death is the first date of the month in each death occurred (this analysis needs to be interpreted in conjunction with the next sensitivity analysis).

***Table E2: regression analysis results***

| **Parameter** | **HR** | **95% CI** | **P** | **HR** | **95% CI** | **P** |
| --- | --- | --- | --- | --- | --- | --- |
| **Gamma** |  |  |  |  |  |  |
| **Sex (female . Male)** | 0.83 | 0.80 , 0.85 | <0.001* | 0.82 | 0.79 , 0.85 | <0.001* |
| **Age at baseline** | 1.09 | 1.08 , 1.11 | <0.001* | 1.64 | 1.60 , 1.67 | <0.001* |
| **Charlson comorbidity index** | 1.03 | 1.02 , 1.04 | <0.001* | 1.23 | 1.21 , 1.24 | <0.001* |
| **Cohort year** | 0.99 | 0.99 , 1.00 | 0.034* | 1.14 | 1.14 , 1.15 | <0.001* |
| **SES (high v. low)** | 0.78 | 0.75 , 0.80 | <0.001* | 1.02 | 0.98 , 1.06 | 0.361 |
| **SES (missing v. low)** | 0.62 | 0.54 , 0.70 | <0.001* | 1.13 | 0.97 , 1.31 | 0.126 |
| **Long length of stay at baseline** | 1.06 | 1.02 , 1.09 | 0.004* | #N/A | #N/A | #N/A |
| **If the patient was admitted at ICU** | 1.06 | 1.01 , 1.11 | 0.011* | #N/A | #N/A | #N/A |
| **Exacerbation 1** | 1.79 | 1.73 , 1.86 | <0.001* | 1.30 | 1.24 , 1.36 | <0.001* |
| **Exacerbation 2** | 1.37 | 1.31 , 1.43 | <0.001* | 0.91 | 0.84 , 0.97 | 0.007* |
| **Exacerbation 3** | 1.19 | 1.13 , 1.25 | <0.001* | 0.89 | 0.80 , 0.99 | 0.030* |
| **Exacerbation 4** | 1.12 | 1.05 , 1.19 | <0.001* | 1.01 | 0.88 , 1.16 | 0.886 |
| **Exacerbation 5** | 1.00 | 0.92 , 1.08 | 0.998 | 0.90 | 0.75 , 1.07 | 0.235 |
| **Exacerbation 6+** | 1.19 | 1.11 , 1.28 | <0.001* | 0.84 | 0.70 , 1.00 | 0.051 |
